# Loss of endothelial glucocorticoid receptor promotes angiogenesis via upregulation of Wnt/β-catenin pathway

**DOI:** 10.1007/s10456-021-09773-x

**Published:** 2021-03-02

**Authors:** Bing Liu, Han Zhou, Tiening Zhang, Xixiang Gao, Bo Tao, Hao Xing, Zhenwu Zhuang, Alan Dardik, Themis R. Kyriakides, Julie E. Goodwin

**Affiliations:** 1 Department of Pediatrics, Yale University School of Medicine, New Haven, CT 06520 USA; 2 Vascular Biology and Therapeutics Program, Yale University School of Medicine, New Haven, CT 06520 USA; 3 Department of Pediatrics, Shengjing Hospital of China Medical University, Shenyang, 110004 China; 4 Department of Vascular Surgery, Xuanwu Hospital, Capital Medical University and Institute of Vascular Surgery, Capital Medical University, Beijing, China; 5 Department of Pharmacology, Yale University School of Medicine, New Haven, CT 06520 USA; 6 Department of Biomedical Engineering, Yale University School of Medicine, New Haven, CT 06510 USA; 7 Section of Cardiovascular Medicine, Yale University School of Medicine, New Haven, CT 06520 USA; 8 Department of Internal Medicine, Yale Cardiovascular Research Center, New Haven, CT 06510-3221 USA; 9 Department of Surgery, Yale University School of Medicine, New Haven, CT 06520 USA; 10 Department of Surgery, VA Connecticut Healthcare Systems, West Haven, CT 06516 USA; 11 Department of Pathology, Yale University, New Haven, CT 06510 USA

**Keywords:** Endothelium, Glucocorticoid receptor, Wnt/β-catenin pathway, Angiogenesis, Autophagy

## Abstract

**Objective:**

The glucocorticoid receptor (GR) is a member of the nuclear receptor family that controls key biological processes in the cardiovascular system and has recently been shown to modulate Wnt signaling in endothelial cells. Wnt/β-catenin signaling has been demonstrated to be crucial in the process of angiogenesis. In the current study, we studied whether GR could regulate angiogenesis via the Wnt/β-catenin pathway.

**Approach and Resultsa:**

Key components of the Wnt/β-catenin pathway were evaluated using quantitative PCR and Western blot in the presence or absence of GR. Enhanced angiogenesis was found in GR deficiency in vitro and confirmed with cell viability assays, proliferation assays and tube formation assays. Consistent with these in vitro findings, endothelial cell-specific GR loss GR in vivo promoted angiogenesis in both a hind limb ischemia model and sponge implantation assay. Results were further verified in a novel mouse model lacking endothelial LRP5/6, a key receptor in canonical Wnt signaling, and showed substantially suppressed angiogenesis using these same in vitro and in vivo assays. To further investigate the mechanism of GR regulation of Wnt signaling, autophagy flux was investigated in endothelial cells by visualizing auto phagolysosomes as well as by assessing P62 degradation and LC3B conversion. Results indicated that potentiated autophagy flux participated in GR-Wnt regulation.

**Conclusions:**

Lack of endothelial GR triggers autophagy flux, leads to activation of Wnt/β-catenin signaling and promotes angiogenesis. There may also be a synergistic interaction between autophagy and Wnt/β-catenin signaling.

**Supplementary Information:**

The online version of this article (10.1007/s10456-021-09773-x) contains supplementary material, which is available to authorized users.

## Introduction

Angiogenesis, defined as the formation of new capillaries from pre-existing blood vessels, is an essential physiological process involved in wound healing, ovulation, vascular and embryonic development [1]. It is a complex process characterized by the migration of endothelial tip and stalk cells in response to a variety of stimuli including chemokines and growth factors such as VEGFA, and is regulated by a variety of signaling pathways [2]. Disordered angiogenesis stimulates a wide range of pathophysiological conditions such as ischemic circulatory disease, cancer, chronic inflammation, atherosclerosis, arthritis and diabetic retinopathy [3]. The development of angiogenesis inhibitors and immunotherapies represents an active area of investigation to combat cancer and other sequelae of dysregulated angiogenesis [4–6]. Despite some advances in this field, angiogenesis inhibitors have, in general, only extended the survival of chemotherapy patients for a few months and resistance to treatment often develops rapidly [7]. Thus, further investigation of underlying molecular mechanisms of angiogenesis is critical to understanding these common disease processes.

The Wnt proteins are a family containing 19 secreted cysteine-rich glycoproteins that accumulate in the extracellular matrix to activate pathways in adjacent cells; they are highly conserved among species [8]. The Wnt/β-catenin pathway is involved in cellular proliferation, survival, differentiation, migration and apoptosis [9, 10]. Moreover, this pathway also appears to be critical in vascular endothelial cells and acts through a variety of regulators, including Bach1 [11], Rspo1 [12], Endostar [13], and ERG [14]. In general, the Wnt/β -catenin pathway is activated when a Wnt ligand binds to its coreceptor complex, which contains a Frizzled family member and low-density lipoprotein receptor-related proteins 5 and 6 (LRP5/6) [15]. As a result, β-catenin binds to the T cell factor/lymphoid enhancer-binding factor (TCF/LEF) family in the nucleus, and alters the expression of key regulators of angiogenesis, such as VEGF, IL-8, Cyclin D1 and MMP-2 [16].

The glucocorticoid receptor (GR) is encoded by the gene *NR3C1*, which is located on chromosome 5q31-32 in humans, and acts as a ligand-inducible transcription factor belonging to the nuclear receptor superfamily [17]. It controls many distinct gene networks, governing various aspects of development, metabolism, inflammation and the stress response, as well as other key biological processes in the cardiovascular system [18]. Glucocorticoids are often components of chemotherapeutic regimens due their recognized angiostatic properties [19]. In our previous work, we have demonstrated that GR is involved in the pathogenesis of atherosclerosis, sepsis and proteinuria [20–22]. Recently, using a genomic approach (GSE119093),as well as in vitro and in vivo studies, we demonstrated that the loss of endothelial GR (eGR) resulted in upregulation of Wnt signaling [23]. These genomic data also revealed that there was enhanced binding of some autophagy-related genes in the absence of GR. Given the interest in better determining the links between autophagy, angiogenesis and cancer [24], we aimed to investigate whether GR could regulate angiogenesis via the Wnt signaling pathway and additionally whether GR could regulate Wnt signaling through autophagy flux.

## Materials and methods

### Reagents

AllStars negative control siRNA and Stealth RNAi for NR3C1 were from Qiagen. CellTiter-Glo® Luminescent Cell Viability Assay (G7570) and CellTiter 96® AQueous One Solution Cell Proliferation Assay (G3582) were purchased from Promega. Matrigel® Basement Membrane Matrix, Growth Factor Reduced (GFR) was purchased from Corning. Antibodies against LRP5 (5731s), LRP6 (3395s), p-β-catenin (4176s), Cyclin D1 (2978s), LC3A/B (4108s) and p62 (5114 s) were purchased from Cell Signaling Technology. Antibodies against α-smooth muscle Actin (α-SMA) (ab5694), CD68 (ab53444), GAPDH (ab8245) and recombinant mouse Wnt3a protein (ab81484) were purchased from Abcam. Antibodies against CD31 (553370) and CD102 (553326) were purchased from BD Bioscience. Anti-GR (sc-393232) was purchased from Santa Cruz Biotechnology. Recombinant mouse sFRP3 protein (592-FR/CF) was purchased from Bio-techne. Rapamycin (tlrl-rap) was purchased from InvivoGen. Premo™ Autophagy Tandem Sensor RFP-GFP-LC3B Kit including chloroquine (P36239) was purchased from Thermo Fisher Scientific.

### Quantitative PCR

MLECs were treated with control or GR siRNA for 48 h. Total RNA was isolated from cells using the RNeasy Mini kit (Qiagen) according to the manufacturer’s instructions. RNA was reverse transcribed using the iScript cDNA Synthesis kit (Bio-Rad) and qPCR was performed on a Bio-Rad C1000 Touch thermal cycler using the resultant cDNA, qPCR Master mix and gene specific primers. The following primers were used:

β-catenin: Forward 5′ TGACACCTCCCAAGTCCTTT 3′.

and Reverse 5′ TTGCATACTGCCCGTCAAT 3′.

TCF4: Forward 5′ GGTGGCCGAATGCACATTGAAAGA 3′.

and Reverse 5′ TTTGCCTGTTCTTCCCTGGACA 3′.

GR: Forward 5′ TGGAGAGGACAACCTGACTTCC 3′.

and Reverse 5′ ACGGAGGAGAACTCACATCTGG 3′.

LRP5: Forward 5′ CTTTCCCCACGAGTATGTTGGT 3′.

and Reverse 5′ AAGGGACCGTGCTGTGAGC 3′.

LRP6: Forward 5′ GGCTGGCATGTGATTGGCT 3′.

and Reverse 5′ GCTCTGGGTTGATCCAACTCT 3′.

Gene expression was normalized to the housekeeping gene 18 s and is presented as fold change.

### Western Blot

Cells were lysed on ice with lysis buffer (50 mM Tris·HCl pH 7.4, 0.1 mM EDTA, 0.1 mM EGTA, 1% Nonidet P-40, 0.1% sodium deoxycholate, 0.1% SDS, 100 mM NaCl, 10 mM NaF, 1 mM sodium pyrophosphate, 1 mM sodium orthovanadate, 1 mM Pefabloc SC, and 2 mg/ml protease inhibitor mixture; Roche Diagnostics). Protein concentrations were determined with the DC Protein assay kit (Bio-Rad Laboratories). Lysates were analyzed by SDS/PAGE and immunoblotted. Primary antibodies used include the following: LRP5, LRP6, phospho-β-catenin, Cyclin D1, GR, LC3A/B, P62 and GAPDH as the loading control. Secondary antibodies were fluorescence-labeled antibodies (LI-COR Biotechnology). Bands were visualized with the Odyssey Infrared LI-COR system (LI-COR Biotechnology).

### Isolation and culture of ECs from murine aorta

For primary mouse aortic endothelial cell (MAOEC) isolation and culture, 10-day-old C57BL/6 (WT), *GR fl/fl, Tie-1 Cre* + (eGR KO) or *LRP5 fl/fl, LRP6 fl/fl, Tie-1 Cre* + (eLRP5/6 KO) mice (*n* = 3/group) were sacrificed by an overdose of isoflurane. The aortas were removed, minced into 1 mm-sized pieces, washed in DMEM medium, and filtered through a 40 µm nylon-mesh cell strainer. The tissue was digested in 15 mL collagenase A for 45 min at 37 °C and the homogenate was further filtered through a 70 µm cell-strainer and centrifuged at 800 rpm for 8 min. The resulting pellet was then washed with phosphate-buffered saline supplemented with 0.1% bovine serum albumin (0.1%BSA/PBS) and the cell suspension was incubated with rat anti-mouse CD31 antibody (553370, BD Bioscience)-coated magnetic beads (sheep anti-rat; 11035, Thermo Fisher Scientific) for 15 min on a Dynalmixer. The magnetic beads were washed four times with PBS/BSA on Dynal-MPC and resuspended in EBM-2 culture medium containing 20% fetal bovine serum (FBS, Cambrex), endothelial cell growth factor with penicillin (100U/ml) and streptomycin (100 g/ml; Invitrogen) and plated on gelatin (Sigma Aldrich)-coated dishes. Cells were re-purified with CD102 antibody (553326, BD Bioscience)-coated magnetic beads during the first two passages and cells from the third passage were used for experiments.

### Cell culture

The MLECs and MAOECs were routinely cultured at 37 °C with 5% CO_2_ in EBM-2 endothelial cell (EC) basal medium with SingleQuot Kits (Lonza, Walkersville, MD) as well as 10% FBS. Cells were used in experiments at passages three to five. For experiments involving Wnt3a or sFRP3 administration, cells were cultured and starved for 4 h in 0.5% FBS. Then they were treated with either recombinant Wnt3a (200 ng/ml) or recombinant sFRP3 (200 ng/ml) for 4 h.

### Cell viability assay

Cell viability was analyzed by CellTiter-Glo® Luminescent Cell Viability Assay (Promega) according to the manufacturer’s instructions. Briefly, 5 × 10^3^ cells/well were seeded in a 96-well plate in 100 μl EBM-2 with 10% FBS/well and we waited 4 h until all the cells became attached. At 4 h and 24 h, 100ul CellTiter-Glo® Reagents were added per well. After mixing contents for 2 min on an orbital shaker and incubating at room temperature for 10 min, luminescence was recorded using a spectrophotometer microplate reader. The assays were carried out in triplicate. Each experiment was repeated three independent times.

### Cell proliferation assay

EC proliferation was analyzed by CellTiter 96® AQueous One Solution Cell Proliferation Assay (Promega) according to the manufacturer’s instructions. Briefly, 5 × 10^3^ cells/well were seeded in a 96-well plate in 100 μl EBM-2 with 10% FBS/well and we waited 4 h until all the cells became attached. At 4 h and 24 h, 20ul CellTiter 96® AQueous One Solution Reagents were added per well. Then, the plate was incubated at 37 °C for 4 h in a humidified, 5% CO_2_ atmosphere. The absorbance was measured at 490 nm using a spectrophotometer microplate reader. The assays were carried out in triplicate. Each experiment was repeated three independent times.

### Tube formation assay

7.5 × 10^4^ cells were cultured in a 24-well plate coated with 250 μl of growth factor-reduced Matrigel (Corning). At 9 h, tubes were photographed in five random microscopic fields with an inverted phase-contrast microscope. Then, the original images were analyzed by the software ImageJ-Angiogenesis Analyzer. In this process a map of the tubes in each imaged was generated and the number of nodes, junctions, branches and length of branches were calculated by ImageJ software. Each experiment was repeated three independent times.

### Mice

*GR fl/fl, Tie-1 Cre* + (eGR KO) mice were generated as previously described [25]. *LRP5 fl/fl, LRP6 fl/fl* mice were the kind gift of Dr. Dianqing Wu at Yale University. These mice were bred to obtain endothelial-specific LRP5/6-deficient (*LRP5 fl/fl, LRP6 fl/fl, Tie-1 Cre* +) (eLRP5/6 KO) mice as well as controls (*LRP5 fl/fl, LRP6 fl/fl, Tie-1 Cre-*). In vivo experiments were performed with eGR KO and eLRP5/6 KO male mice and their wild-type (WT) littermates aged 8–12 weeks. All mice were housed in a standard, pathogen-free facility. All studies were approved by the IACUC at Yale University School of Medicine, New Haven, Connecticut, USA, and were consistent with the *Guide for the Care and Use of Laboratory Animals* (National Academies Press, 2011).

### Hind limb ischemia model

The severe hind limb ischemia (HLI) model was conducted under anesthesia with isoflurane delivered via a precision vaporizer at 2%. First, the right femoral artery was exposed under a dissection microscope. The proximal femoral artery and the distal portion of the saphenous artery were ligated and arteriectomy was performed. No operation was performed on the left femoral artery, which was regarded as the healthy control. Blood flow recovery to the ischemic foot was sequentially monitored by a MoorLDI2™ laser Doppler imaging system (Moor Instruments, Devon, UK) for up to 21 days after ischemia. Mice were anesthetized and maintained at 37 °C on a heating plate to minimize temperature variation. Ischemic and non-ischemic limb perfusion was measured pre- (day 0), and post-surgery at days 3,7,14 and 21. Final blood flow values were presented as the ratios of ischemic to non-ischemic hindlimb perfusion. Mice were sacrificed on day 21 post-surgery and adductor muscles were harvested, hematoxylin and eosin (H&E) staining was performed to demonstrate the morphology of the muscle tissue, and immunostaining was performed to investigate capillary density, arteriolar density and macrophage density. Capillaries in tissue sections were stained using a rat anti-mouse monoclonal CD31 antibody and arterioles were visualized using a rabbit anti-mouse monoclonal α-smooth muscle actin (α-SMA) antibody. Macrophages were also recognized by a rat anti-mouse monoclonal CD68 antibody. Donkey anti-rat IgG (Alexa Fluor® 488) and donkey anti-rabbit IgG (Alexa Fluor® 488) were used as secondary antibodies for visualization. CD31-positive, α-SMA-positive and CD68-positive areas were quantified in randomly acquired images using ImageJ. Three mice were used in each group.

### Sponge implantation assay

For sponge implantation experiments, animals were anesthetized via isoflurane delivered from a precision vaporizer at 2%. A single midline incision was made and two sub-dermis implantation sites were created by blunt dissection on each side of the incision. 9 mm-dimension polyvinyl alcohol (PVA) sponges (Henry Schein) were implanted in the corresponding sites. Ten days later, sponges were excised and made into cryosections, then they were stained with anti-CD31 antibody, anti-α-SMA antibody or anti-CD68 antibody and visualized. Six mice were used in each group.

### Autophagy assessment

MLECs were cultured as mentioned above. For autophagy induction, cells were washed three times with PBS and incubated for 4 h in Hanks balanced salt solution (HBSS; Invitrogen) or complete medium containing 2 μM rapamycin (Rap) at 37 °C. Chloroquine (CQ) was used as an autophagy inhibitor at a concentration of 30 μM for 16 h in the complete medium. In some experiments, Wnt3a was administrated for 4 h after treatment with Rap or CQ. Autophagy was assessed by Premo™ Autophagy Tandem Sensor RFP-GFP-LC3B Kit, LC3II/I conversion and p62 degradation. For visualization of autophagosomes and auto phagolysosomes, cells were fixed with 4% formaldehyde and permeated with 0.1% Triton X-100 after incubation with the RFP-GFP-LC3 Kit, then an immunofluorescent microscope was used to visualize the images.

### Statistical analysis

To investigate the influence of gene defect, binary comparisons (siCT vs siGR, WT vs eGRKO, eGRKO vs eLRP5/6KO, WT vs eLRP5/6KO) were analyzed using Student’s 2-tailed t tests. To study the effect of certain signaling pathway by using agonists and antagonists, multiple comparisons were analyzed using One-way ANOVA with Tukey’s post hoc test (Control vs Wnt3a vs sFRP3, Control vs Dex vs RU486, Control vs HBSS vs CQ, Control vs Rap vs CQ). Data are expressed as mean ± SEM. A p value less than 0.05 was considered significant. GraphPad Prism 8 was used to generate graphs and perform statistical analysis.

## Results

### Loss of GR up regulates the canonical Wnt/β-catenin pathway in vitro.

**Fig. 1:**
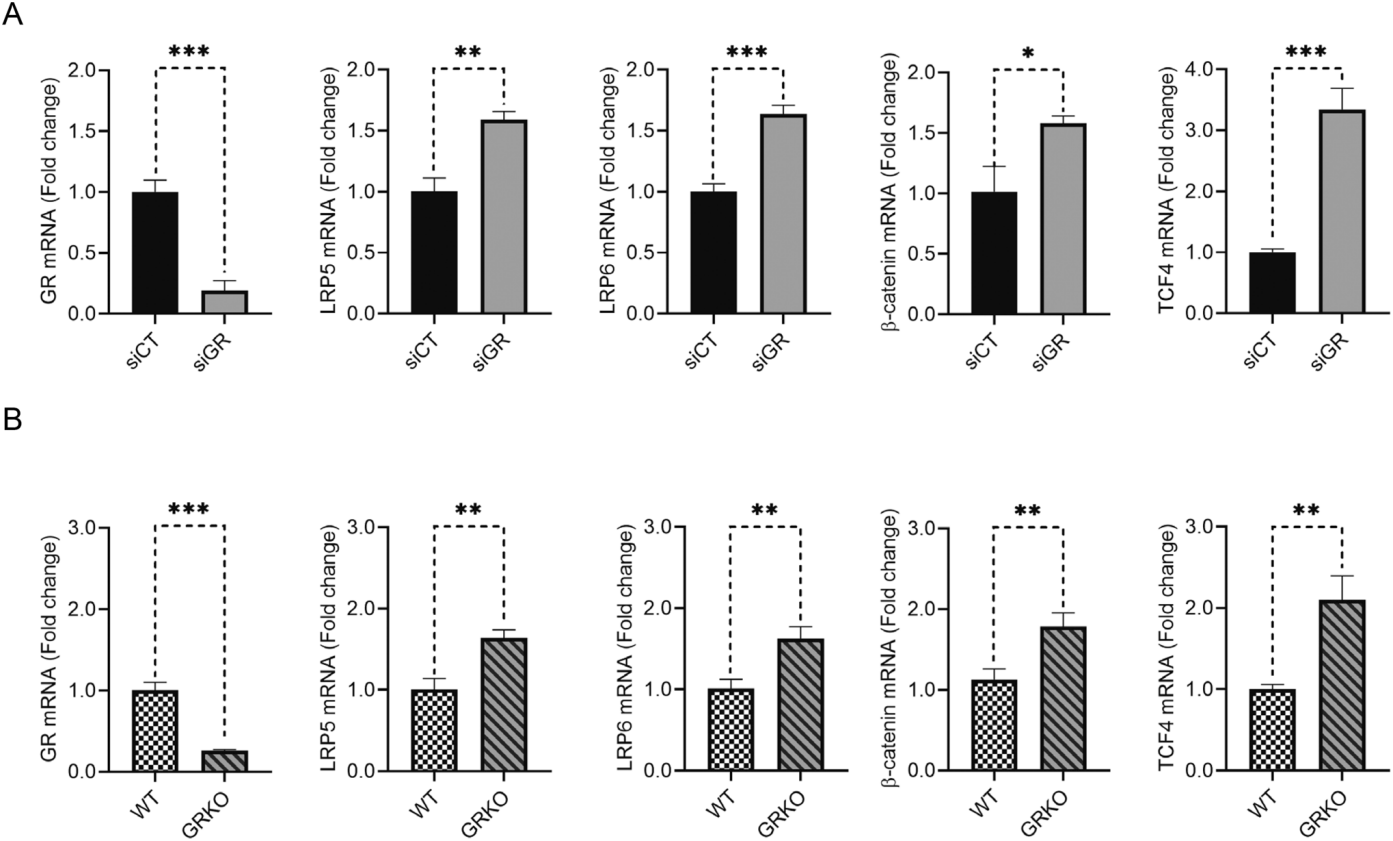
Loss of GR upregulates the canonical Wnt/β-catenin pathway at the mRNA level. **a** MLECs transfected with GR siRNA had higher expression of LRP5, LRP6, β-catenin and TCF4 than those transfected with control siRNA (*t*-test, *n* = 3/group), **b** Primary MAOECs from eGRKO mice had higher expression of LRP5, LRP6, β-catenin and TCF4 than those from their WT littermates (*t*-test, *n* = 3/group) *P < 0.05, **P < 0.01, ***P < 0.001. *siCT* MLECs transfected with control siRNA, *GRsi* MLECs transfected with control siRNA, *WT* primary MAOECs from WT mice, *GRKO* primary MAOECs from eGRKO mice

To investigate whether GR acts as a negative regulator of the Wnt/β-catenin pathway in MLECs, we transfected MLECs with either control or GR siRNA and then examined the expression of key components in the Wnt/β-catenin pathway by qPCR. As presented in Fig. 1a, the mRNA expression of LRP5, LRP6, β-catenin and TCF4 increased as a result of GR knockdown, indicating that loss of GR resulted in the up regulation of the Wnt signaling pathway at the mRNA level. In contrast to GR-replete cells, GR-deficient MAOECs also had higher mRNA expression of LRP5, LRP6, β-catenin and TCF4 (Fig. 1b). MLECs were also stained with antibodies against β-catenin and GR and showed that GR deficiency contributed to the accumulation of β-catenin in the nucleus (Suppl Fig. S1a and S1b). This is in agreement with our previously published results [23].

**Fig. 2:**
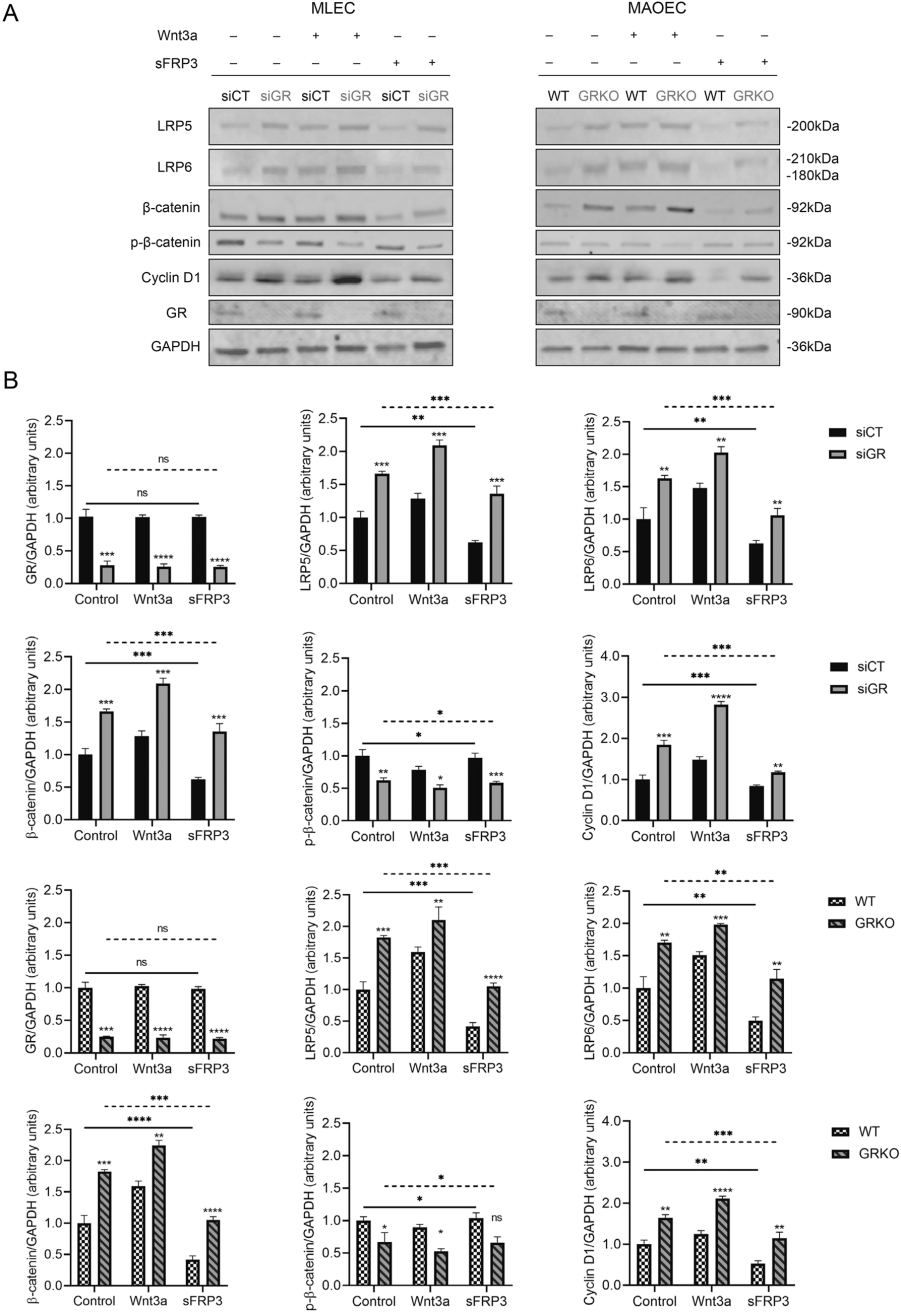
GR negatively regulates the canonical Wnt/β-catenin proteins. **a** Representative western blot bands demonstrate that the expression of LRP5, LRP6, β-catenin and Cyclin D1 is increased, while the expression of phospho-β-catenin is decreased in GR-deficient MLECs and MAOECs (*t*-test). Treatment with Wnt3a promotes the activation of Wnt signaling, while sFRP3 treatment can diminish this effect in vitro (One-way ANOVA). **b** Quantitative results of western blots presented as bar graphs (*n* = 3/group, *P < 0.05, **P < 0.01, ***P < 0.001, ****P < 0.0001, *ns* not significant). *siCT* MLECs transfected with control siRNA, *GRsi* MLECs transfected with control siRNA, *WT* primary MAOECs from WT mice, *GRKO* primary MAOECs from eGRKO mice

Next, we evaluated the protein expression of LRP5, LRP6, phospho-β-catenin and Cyclin D1. As demonstrated in Fig. 2a and quantified in 2b, in GR siRNA-treated MLECs as well as primary MAOECs from eGR KO mice, the levels of LRP5 and LRP6 were significantly increased compared to eGR-replete conditions. The expression of phospho-β-catenin, the inactive isoform of β-catenin, decreased in GR deficient cells. Cyclin D1, which is an important angiogenic regulator, has been reported to be regulated by Wnt/β-catenin pathway [26]. Therefore, we also assessed the expression of this gene and observed that the level of Cyclin D1 increased as a result of GR knockdown. Treatment with Wnt3a, a canonical Wnt ligand, was able to regulate such effects positively while the administration of sFRP3, a Wnt antagonist, could suppress the expression of LRP5, LRP6 and Cyclin D1 in both MLECs and MAOECs.

### GR influences angiogenesis in vitro by regulating the canonical Wnt/β-catenin pathway.

**Fig. 3:**
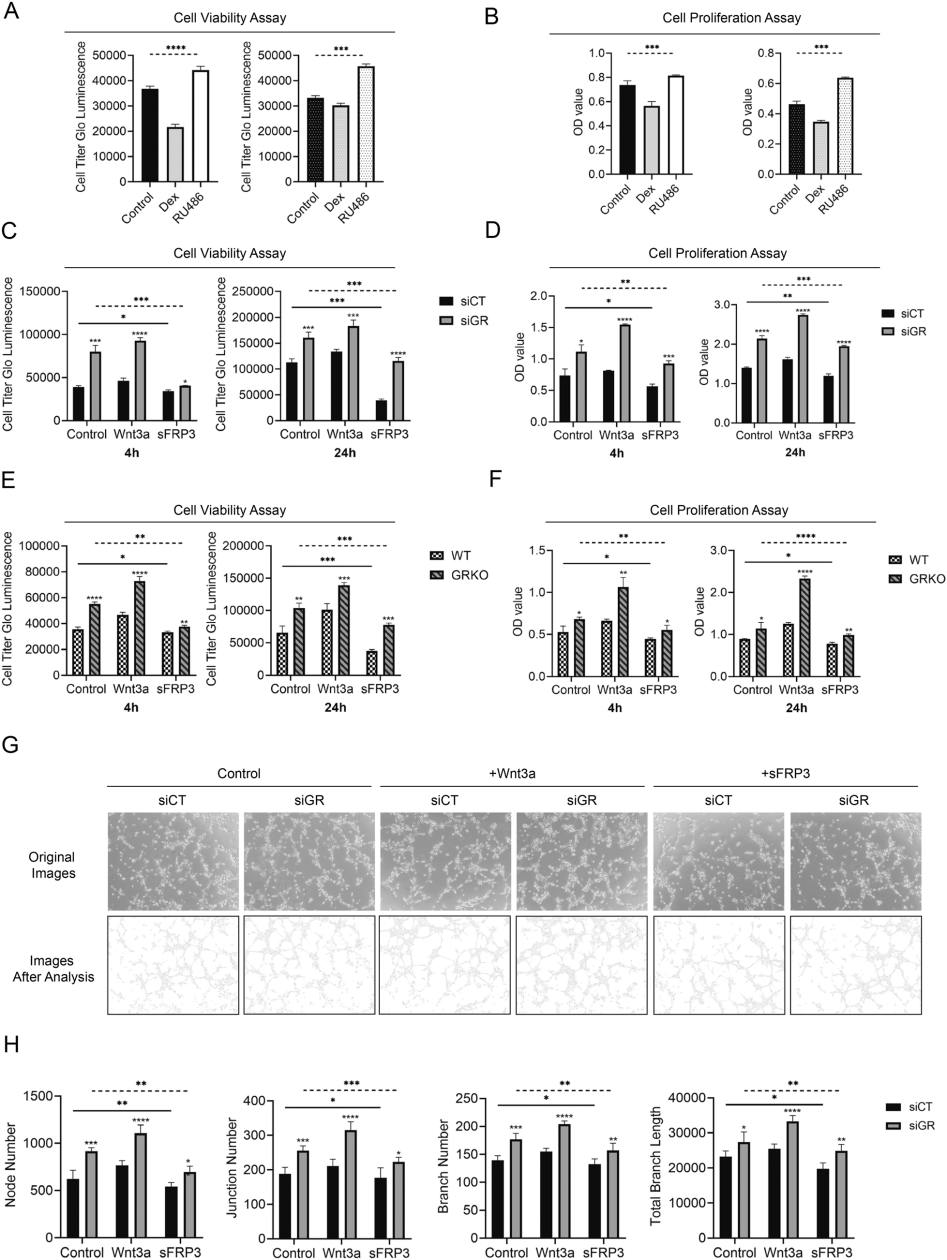
GR influences angiogenesis in vitro by regulating Wnt/β-catenin pathway. **a** Treatment with Dex suppressed cell viability, while RU486 had the opposite effect in MLECs (left) and MAOECs (right) (One-way ANOVA). **b** Dex inhibited cell proliferation, while RU486 increased proliferation in MLECs (left) and MAOECs (right) (One-way ANOVA). **c** Loss of GR promoted cell viability at both 4 h and 24 h in MLECs (*t*-test). Wnt3a could enhance this effect while sFRP3 had an inhibitory effect (One-way ANOVA) (*n* = 3/group). **d** GR knockdown enhanced cell proliferation via the Wnt/β-catenin pathway at both 4 h and 24 h in MLECs and MAOECs (*t*-test). Wnt3a treatment enhanced this effect while sFRP3 negatively affected cell proliferation (One-way ANOVA) (*n* = 3/group). **e** Loss of GR promoted cell viability at both 4 h and 24 h in MAOECs (*t*-test). Wnt3a could enhance this effect while sFRP3 had an inhibitory effect (One-way ANOVA) (*n* = 3/group). **f** GR knockdown enhanced cell proliferation via the Wnt/β-catenin pathway at both 4 h and 24 h in MAOECs (*t*-test). Wnt3a treatment enhanced this effect while sFRP3 negatively affected cell proliferation (One-way ANOVA) (*n* = 3/group). **g** Original tube formation images and corresponding tube maps. **h** Tube formation assay demonstrated that MLECs transfected with GR siRNA had more nodes, junctions and branches than MLECs transfected with control siRNA (*t*-test). The introduction of Wnt3a promoted the formation of tubes, while sFRP3 had the opposite effect (One-way ANOVA) (*n* = 5/group). *P < 0.05, **P < 0.01, ***P < 0.001, ****P < 0.0001

To investigate how GR influenced angiogenesis, we first assessed its effects on the viability and proliferation of endothelial cells by using the GR agonist dexamethasone (Dex) and the GR antagonist RU486. As demonstrated in Fig. 3a, b, after 4 h, both cell viability and proliferation were suppressed with the introduction of 100 nM Dex, while RU486 (100 μM) treatment promoted viability and proliferation in MLECs as well as MAOECs. Therefore, we concluded that GR could independently influence angiogenesis in vitro. Given the well-recognized effects of augmented Wnt signaling on angiogenesis [16], we assessed whether loss of GR could influence the angiogenic phenotype via Wnt signaling in endothelial cells. When cell viability was assessed, as shown in Fig. 3c, MLECs exhibited superior viability in the GR siRNA-treated cell group in contrast to those in the control siRNA-treated cell group, both at 4 h and 24 h. Administration of Wnt3a increased viability while the introduction of sFRP3 reduced viability, indicating the specificity of the observed responses was mediated through Wnt signaling pathway. Similar results were observed using primary MAOECs (Fig. 3e).

Next, cell proliferation was examined. MTS assay demonstrated that the proliferation of GR-deficient MLECs was enhanced (Fig. 3d). Wnt3a was able to augment proliferation in all groups, while treatment with sFRP3 resulted in loss of proliferation. Similar results were obtained from primary MAOECs (Fig. 3f).

Tube formation assay was also conducted to visualize angiogenesis in vitro. In GR- deficient MLECs, there were more nodes, junctions and branches and longer branch length than in control cells. These differences became more evident in the presence of Wnt3a, and less pronounced after the administration of sFRP3 (demonstrated in Fig. 3e,quantified in 3f). We achieved similar results using this assay in primary MAOECs (demonstrated in Suppl Fig. S2a and quantified in Suppl Fig S2b) suggesting that GR suppresses angiogenesis in vitro by tonic inhibition of the canonical Wnt/β-catenin pathway.

To further investigate these phenotypes on a genetic background of impaired Wnt signaling we generated endothelial-cell specific LRP5/6 knockout mice. After verifying that the knockdown of endothelial LRP5/6 was successful (Suppl Fig. S3a), we compared tube formation (Suppl Fig. S2c, d) and the cell viability and proliferation (Suppl. Fig S3b, c) among eGR KO MAOECs, eLRP5/6 KO MAOECs and WT MAOECs and showed that angiogenesis was substantially down-regulated in eLRP5/6 KO cells compared to cells in both other groups.

### Angiogenesis is enhanced in endothelial GR-deficient mice after ischemic injury

To determine whether GR could regulate angiogenesis after ischemic injury, hind-limb ischemia was surgically induced in eGR KO mice and WT littermates. Then blood flow was measured by laser doppler at the following timepoints: one day before surgery (Pre-), day of surgery (day 0), 3 days after surgery (day 3), 7 days after surgery (day 7), 14 days after surgery (day 14) and 21 days after surgery (day 21). From day 7, blood flow in ischemic limbs started to recover faster in eGR KO mice compared to WT controls (Suppl Fig. S4a, b).

**Fig. 4:**
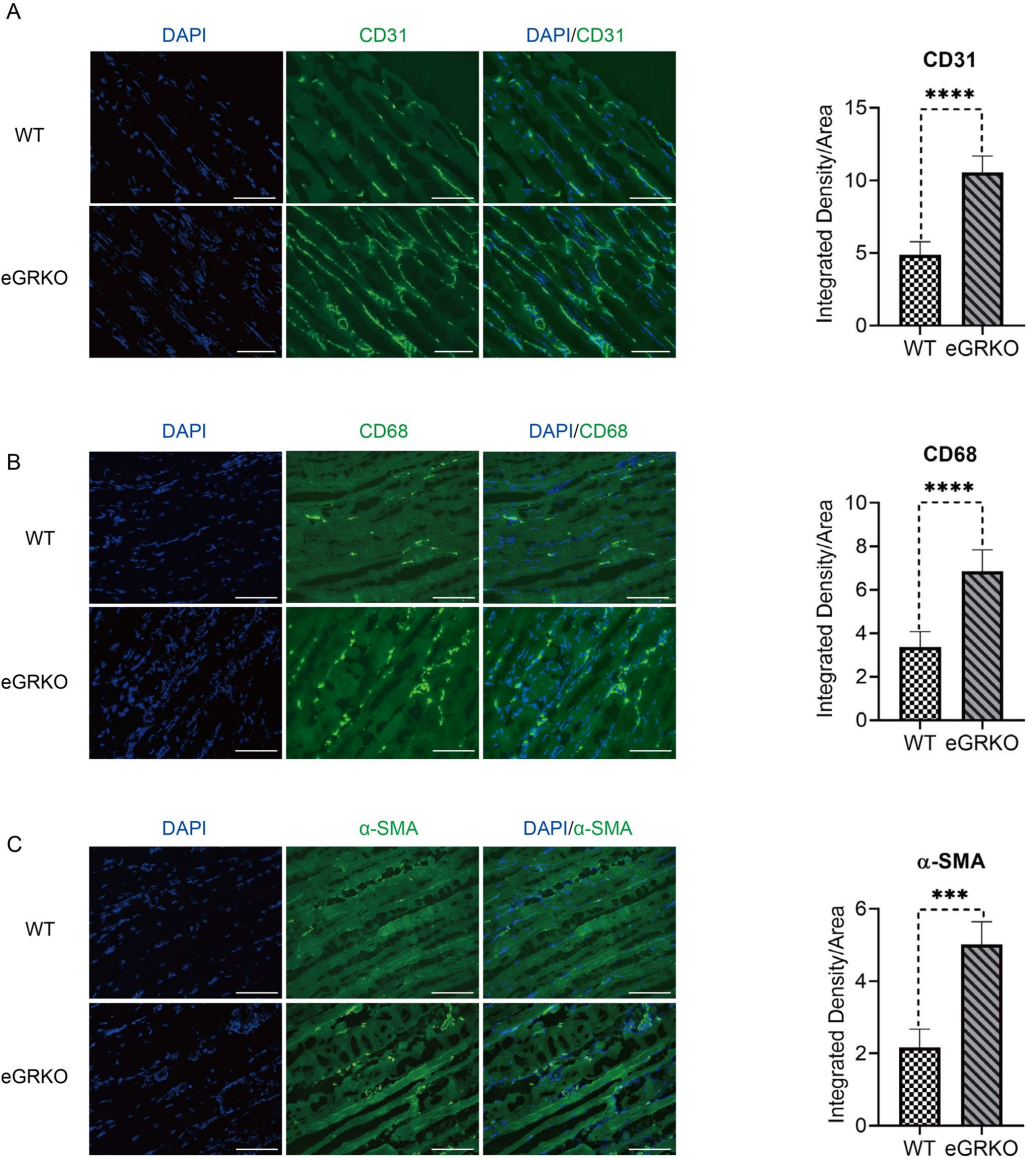
Angiogenesis is enhanced in endothelial GR deficient mice after hind limb ischemia. **a** Representative images of CD31 staining in injured adductor muscles (scale bar, 100 µm); quantitative results demonstrate endothelial GRKO mice have higher expression of CD31. **b** Representative images of CD68 staining in injured adductor muscles (scale bar, 100 µm); quantitative results demonstrate endothelial GRKO mice have higher expression of CD68. **c** Representative images of α-SMA staining in injured adductor muscles (scale bar, 100 µm); quantitative results demonstrate endothelial GRKO mice have higher expression of α-SMA. (*t*-test, *n* = 3/group, ***P < 0.001, ****P < 0.0001)

In addition, at day 21 post-surgery, adductor muscles from the ischemic legs were excised for both H&E staining and immunostaining. As shown in Suppl Fig S4c, there were more necrotic myocytes in muscles from WT mice compared to those from eGRKO mice, which was consistent with the results from laser doppler measurement. Furthermore, eGR deficiency was associated with a marked rise in capillary density, arteriolar density and macrophage density (Fig. 4a-c). Compared to WT mice, eGRKO mice contained more CD31, α-SMA and CD68 staining in ischemic adductor muscles after 21 days, indicating endothelial GR knockout could enhance angiogenesis after ischemic injury.

### Endothelial GR deficiency promotes angiogenesis while endothelial LRP5/6 deficiency suppresses angiogenesis in vivo

**Fig. 5:**
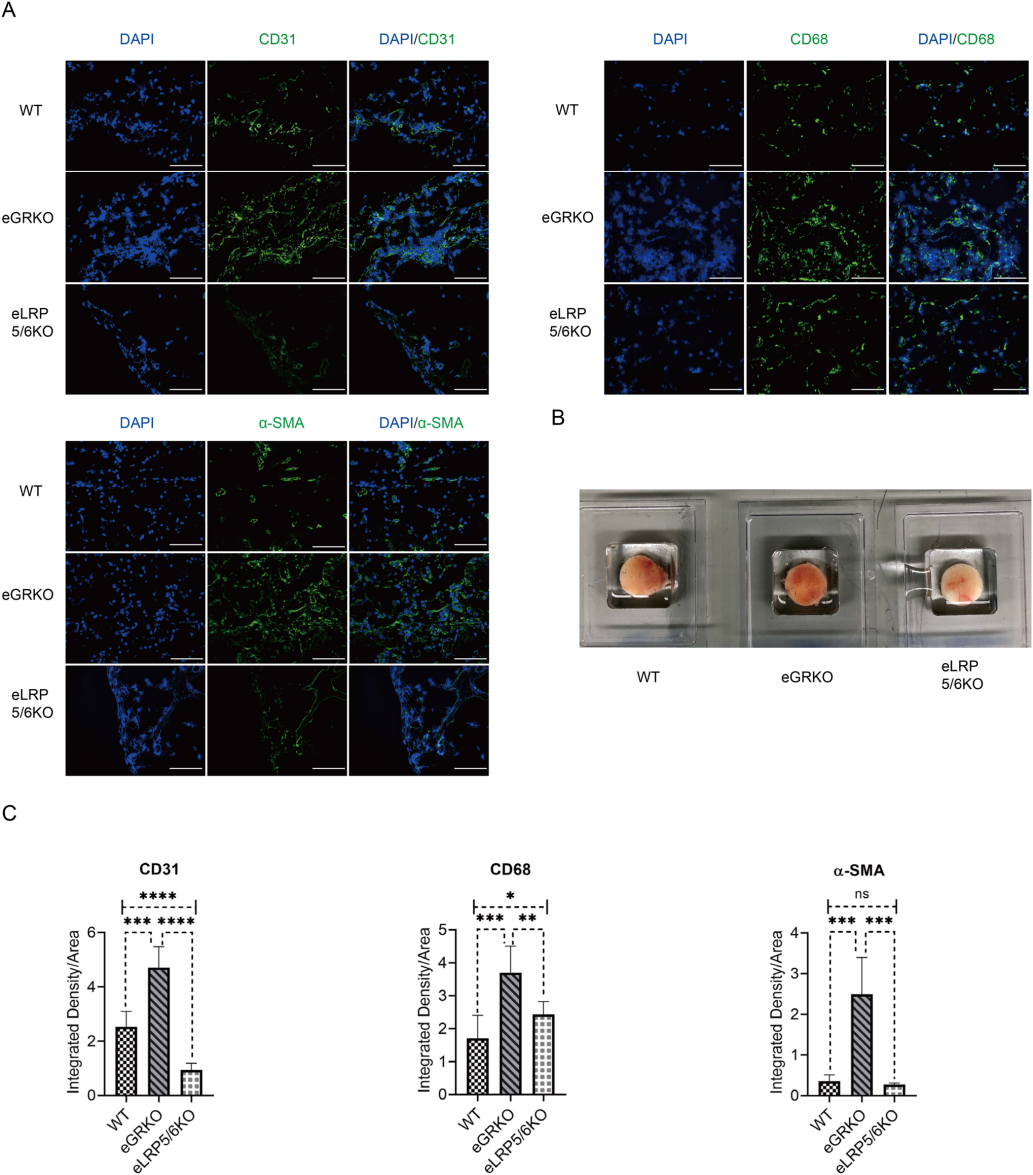
In vivo sponge Implantation assay demonstrates angiogenic phenotype involves canonical Wnt signaling. **a** Representative sponge sections stained with CD31, CD68 and α-SMA from WT mice, eGRKO mice and eLRP5/6KO mice (scale bar, 100 µm). **b** Representative ex vivo sponges after removal from mice of each genotype. **c** Compared to WT mice, eGRKO mice had more CD31 staining while eLRP5/6 mice had less CD31 staining. Both CD68 staining and α-SMA staining were significantly increased in eGRKO mice and suppressed in eLRP5/6KO mice (*t*-test, *n* = 6/group, *P < 0.05, **P < 0.01, ***P < 0.001, ****P < 0.0001, *ns* not significant)

A sponge implantation assay was also performed to assess the angiogenic phenotype. In this model, eGR KO mice, eLRP5/6 KO mice and WT littermates were used. Ten days after subcutaneous implantation, PVA sponges (Fig. 5b) were excised and evaluated for capillary density, arteriolar density and macrophage density by immunofluorescence staining. As presented in Fig. 5a and quantified in Fig. 5c, there was more CD31, CD68 and α-SMA staining in eGR KO mice compared to WT mice, indicating that loss of endothelial GR could stimulate angiogenesis in vivo. To verify the specificity of the Wnt signaling pathway in mediating this effect, we also assessed the sponges implanted in eLRP5/6 KO mice. Immunofluorescence staining of these sponges demonstrated that there was less CD31 and α-SMA staining in eLRP5/6 KO mice than in WT mice. The expression of CD68 was augmented in eLRP5/6 KO mice compared to WT, though it was still lower than in eGR KO mice.

### GR regulates the canonical Wnt/β-catenin pathway via modulation of autophagy flux

**Fig. 6:**
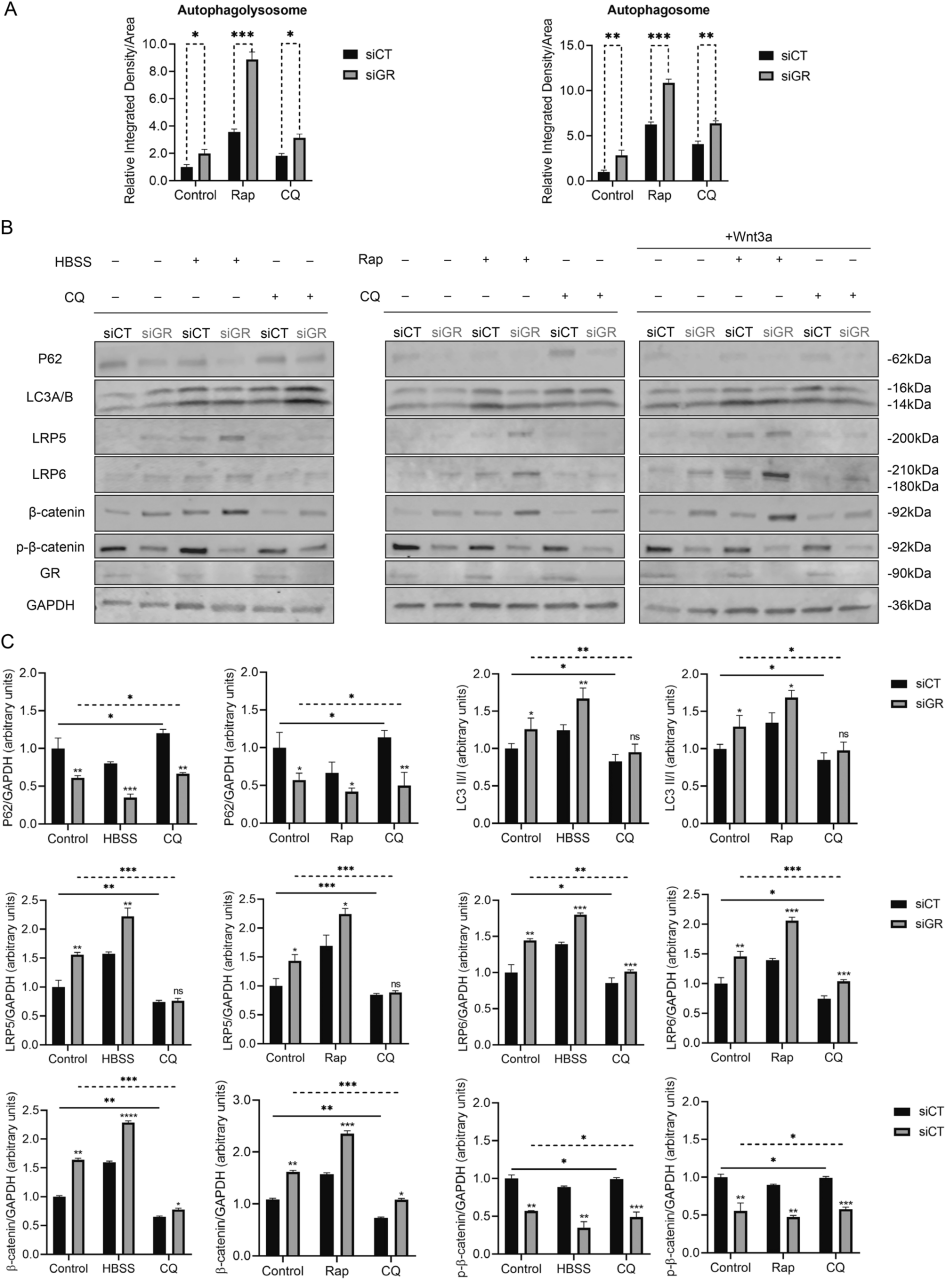
Loss of GR up regulates Wnt/β-catenin pathway by enhancing autophagy flux. **a** Quantitative results indicate that the number of autophagolysosomes increased with GR knockdown, implying loss of GR enhanced autophagy flux (*t*-test). Rapamycin could increase the number of both autophagolysosomes and autophagosomes; however, after treatment with chloroquine, the number and the ratio of autophagolysosomes was smaller than that from cells treated with rapamycin, indicating that autophagy flux was blocked. (One-way ANOVA) (*n* = 5/group). **b** Representative western blots from MLECs treated with either HBSS (4 h), rapamycin (4 h), chloroquine (16 h) or Wnt3a (4 h). Rap: rapamycin; CQ: chloroquine. **c** Western blot densitometry of HBSS/CQ and Rap/CQ treatment conditions (*n* = 3/group). Both HBSS starvation and rapamycin treatment could upregulate Wnt/β-catenin pathway, while chloroquine suppressed the expression of Wnt-related components (One-way ANOVA). *P < 0.05, **P < 0.01, ***P < 0.001, ****P < 0.0001, *ns* not significant. *siCT* MLECs transfected with control siRNA, *GRsi* MLECs transfected with control siRNA

To evaluate whether GR had could influence autophagy flux, MLECs transfected with either control or GR siRNA were used. Cells were treated either with 2 μM rapamycin for 4 h to activate autophagy flux or chloroquine for 16 h to reduce auto phagolysosome formation, as a sign of autophagy inhibition. The Premo™ Autophagy Tandem Sensor RFP-GFP-LC3B kit was used to visualize the change in autophagy flux. In this way, autophagosomes (which at neutral pH display both the GFP and RFP) appear yellow, and autophagolysosomes (which at acidic pH display only the RFP) appear red and can be distinguished (Suppl Fig. S5a). As shown in Fig. 6a, in the absence of rapamycin or chloroquine administration, the autophagy flux in GR-deficient cells was significantly enhanced compared to that in GR-replete cells. Rapamycin could augment both autophagosomes and auto phagolysosomes more efficiently as a result of GR knockdown. However, after treatment with chloroquine, the number of autophagolysosomes and the ratio of autophagolysosomes was smaller than that from cells treated with rapamycin, which indicated that autophagy flux was blocked.

According to our previously generated genomic data (GSE 119092, GSE119093), the autophagy pathway seemed to be differentially regulated by GR; therefore, we tested whether loss of endothelial GR activated the Wnt/β-catenin pathway via enhancing autophagy flux. MLECs transfected with control or GR siRNA were incubated in serum-free HBSS or complete medium containing 2 μM rapamycin for 4 h to induce autophagy; CQ was used as an autophagy inhibitor. As demonstrated in Fig. 6b and quantified in Fig. 6c, GR deficiency resulted in decreased p62 expression and enhanced LC3A/B II/I conversion, consistent with the results from the RFP-GFP-LC3B assay. Additionally, both HBSS incubation and rapamycin treatment were able to increase the expression of LRP5 and LRP6 and decrease the expression of phospho-β-catenin, while CQ had the opposite effects. These data strongly argue that autophagy can promote the activation of the Wnt/β-catenin pathway. Furthermore, we also show that treatment with Wnt3a, a canonical Wnt ligand, could not only up-regulate the Wnt/β-catenin pathway, but also enhance LC3A/B II/I conversion and p62 degradation, implying there is a positive feedback loop between autophagy and Wnt signaling (blots shown in Fig. 6b and quantified in Suppl Fig. S5b).

## Discussion

The major finding of this study is that absence of endothelial GR can augment angiogenesis through activation of the Wnt/β-catenin signaling pathway and that, mechanistically, this occurs via induction of autophagy flux.

Angiogenesis is involved in the development of many diseases. Most importantly, it is regarded as one of the hallmarks of cancer [27]. Dysregulation of angiogenesis in cancer results in an abnormal vascular network, which not only reduces the therapeutic efficacy of conventional anticancer therapies but stimulates tumor progression as well [28]. It is also involved in sequelae of diabetes in that pathological angiogenesis is the main cause of proliferative diabetic retinopathy (PDR), which is the advanced form of diabetic retinopathy (DR). In order to interfere with pathological angiogenesis, multiple treatment approaches have been established, such as gene therapy, monoclonal antibodies (Bevacizumab), miRNAs, small molecules (Sorafenib), Angiostatin and Endostatin [1].

In the current study, we demonstrate that endothelial GR inhibits angiogenesis via suppression of canonical Wnt signaling. When canonical Wnt signaling is up regulated, due to loss of endothelial GR, cell viability, cell proliferation and tube formation ability are all augmented significantly in GR- deficient MLECs and MAOECs, indicating that endothelial GR can tonically suppress both angiogenic functions and the Wnt/β-catenin pathway under homeostatic conditions. According to the results from both the hind limb ischemia model and sponge implantation model, endothelial GR suppresses angiogenesis as well as inflammation in vivo; we have previously demonstrated that endothelial GR acts as a negative regulator of inflammation [21].

Previously, we showed that endothelial GR could bind to TCF/LEF directly [23]; however, here we investigated whether GR could regulate Wnt signaling via other indirect mechanisms. We investigated the expression of p62 between GR-replete and GR-deficient endothelial cells. Intriguingly, we observed an apparent decrease of p62 in GR knockdown cells. As p62 is regarded as a very critical marker of autophagy [29], further analysis focused on whether GR could regulate autophagy flux. By assessing the LC3 II/I ratio in vitro, which was consistent with decreased expression of p62 in GR knockdown cells, we concluded that endothelial GR could regulate autophagy flux. These results were further confirmed by using rapamycin to demonstrate increased formation of both autophagosomes and auto phagolysosomes in the absence of GR. GR has been reported to modulate autophagy in other cell types such as hepatic cells and prostate cancer cells [30, 31], but has not previously been investigated in endothelial cells.

In the absence of GR, rapamycin could also potentiate the protein levels of some key components of Wnt signaling, while CQ had an inhibitory effect. These results indicated that loss of endothelial GR could trigger autophagy flux and augmentation of the Wnt/β-catenin signaling pathway. Administration of Wnt3a seemed to have a positive feedback effect on autophagy according to the expression of p62 and LC3. We speculate there might be a GR-mediated autophagy-Wnt feedback loop which promotes angiogenesis.

The relationship between autophagy and Wnt signaling is somewhat controversial. In some cancer cells, the Wnt pathway component DVL2 maintains glioblastoma multiforme (GBM) cell proliferation through canonical and noncanonical Wnt signaling[32, 33]. In turn, autophagy negatively regulates Wnt signaling by promoting DVL2 and β-catenin degradation[34–36]. However, some other studies reveal different effects. In the study by Ma et al.[37], the authors found that inhibiting autophagy by downregulating Atg5 gene expression impaired hepatic differentiation of hepatic progenitor cells (HPCs) and inhibited activation of the Wnt/β-catenin pathway, which could be rescued by overexpression of the β-catenin gene. Collectively, these results demonstrated that autophagy promotes HPCs differentiation by up-regulating Wnt/β-catenin signaling. In another study[38], researchers examined β-catenin phosphorylation at Ser33 in hepatocellular carcinoma cells cultured with Earle's Balanced Salt Solution (EBSS) or 3- Methyladenine (3-MA) for 6 h. The starved cells had decreased β-catenin phosphorylation, while the autophagy-inhibited cells had increased β-catenin phosphorylation. Immunofluorescence examination of the cellular localization of β-catenin showed that autophagy increased β-catenin nuclear accumulation after 6 h starvation. These results indicated that starvation-induced autophagy could activate β-catenin and were consistent with our current findings in endothelial cells. Additionally, according to the study of Jing et al.[39], in squamous cell carcinoma of the head and neck (SCCHN), Wnt3a binding to its receptor on the membrane activated the canonical Wnt signaling pathway, accelerated the nuclear translocation of β‐catenin and then enhanced the expression of Beclin1, which promoted autophagy. In our study, we also demonstrated that Wnt3a had a positive effect on autophagy.

Our current study also has some limitations. First, in our HLI model, the blood flow in the injured limb recovered to just 20–30% after 21 days, which was lower than that reported in some other studies[40, 41]. However, we noted via direct observation that the ambulation of the mice became normal within 10 days after surgery and the staining of CD31 and α-SMA in the adductor muscles also revealed adequate vascular supply at day 21 after surgery. Second, although we verified GR-deficiency induced autophagy, we didn’t fully investigate the intrinsic mechanism, which will be the subject of a future study.

In conclusion, endothelial GR impedes autophagy flux, leads to suppression of Wnt/β-catenin signaling and, in turn, suppresses angiogenesis. Our data might prove useful in the clinical arena and suggest that autophagy inhibitors or canonical Wnt signaling inhibitors might be used in suppressing angiogenesis and may be as efficacious as steroids, which are fraught with poorly tolerated side effects. With endothelial cell heterogeneity now recognized as a key driver of vascular physiology, both homeostatic and pathological, endothelial GR may be an important therapeutic target with profound effects on specific vascular beds, an innovative concept that has yet to be studied.

## Supplementary Information

Below is the link to the electronic supplementary material.

Supplementary material 1 (PDF 561 kb)

Supplementary material 2 (PDF 801 kb)

Supplementary material 3 (PDF 462 kb)

Supplementary material 4 (PDF 641 kb)

Supplementary material 5 (PDF 836 kb)

Supplementary material 6 (DOCX 16 kb)

## Abbreviations

GR: Glucocorticoid receptor
LRP5/6: Low-density lipoprotein receptor-related proteins 5 and 6
MLEC: Mouse lung endothelial cell
MAOEC: Mouse aorta endothelial cell
HLI: Hind limb ischemia
DAPI: 4,6-Diamidino-2-phenylindole
α-SMA: α-Smooth muscle actin
LC3A/B: Microtubule-associated protein 1A/1B-light chain 3
HBSS: Hanks balanced salt solution
Rap: Rapamycin
CQ: Chloroquine
VEGF: Vascular endothelial growth factor A
