# Supplementary figures and images for: Loss of endothelial glucocorticoid receptor promotes angiogenesis via upregulation of Wnt/β-catenin pathway
Source: Angiogenesis. 2021 Mar 2;24(3):631–45. doi: 10.1007/s10456-021-09773-x (PMC8292305; doi:10.1007/s10456-021-09773-x)

A

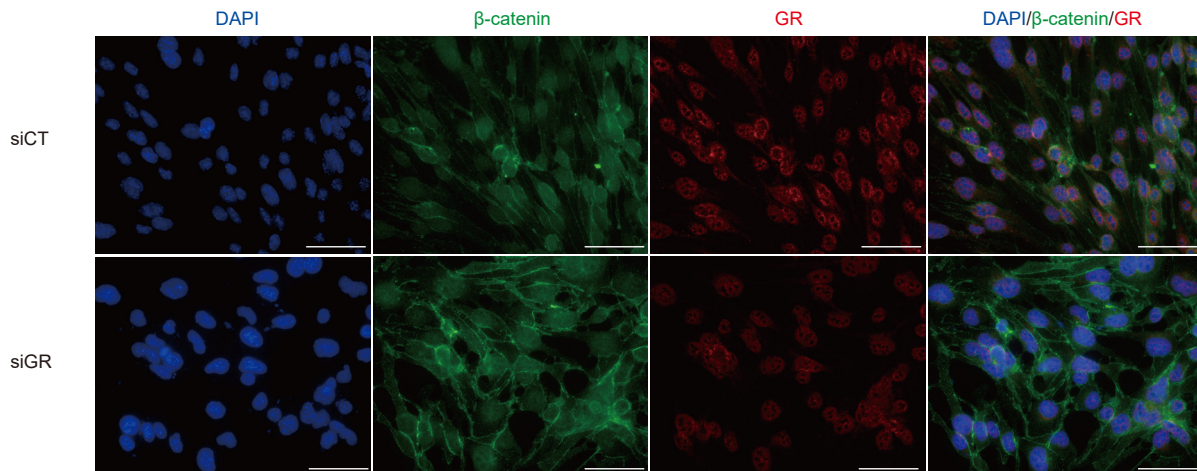

B

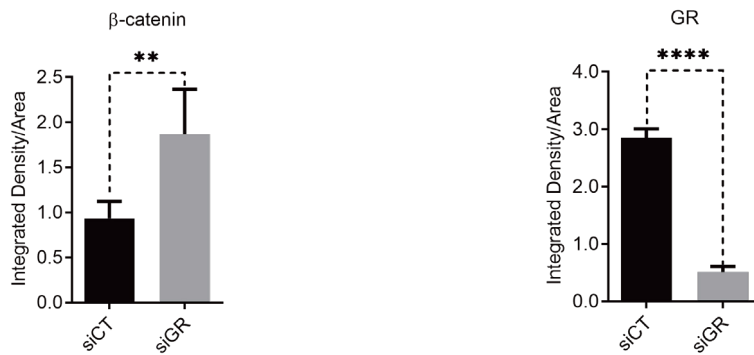

Supplement: Supplementary file 1 — Supplementary material 1 (PDF 561 kb) [file 10456_2021_9773_MOESM1_ESM.pdf]

A

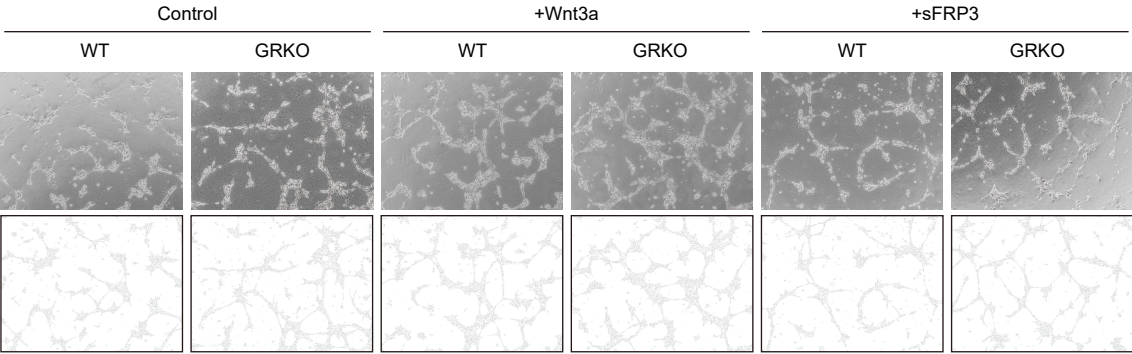

B

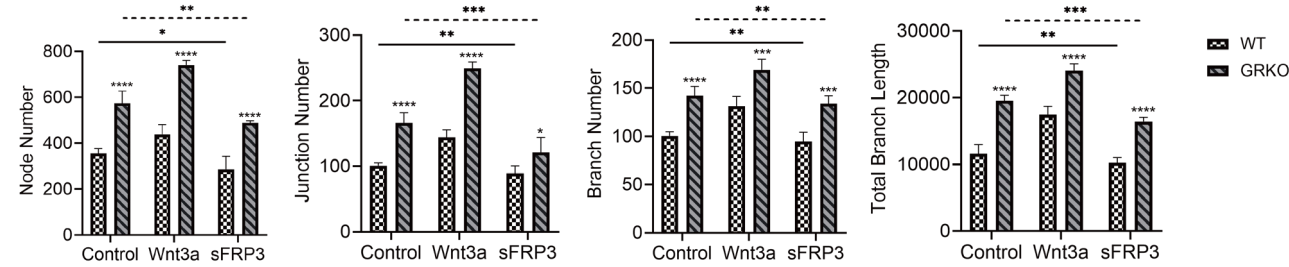

C

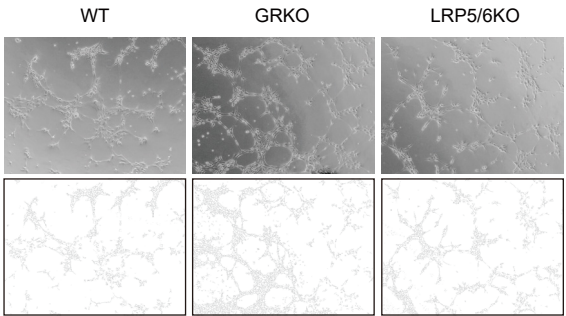

D

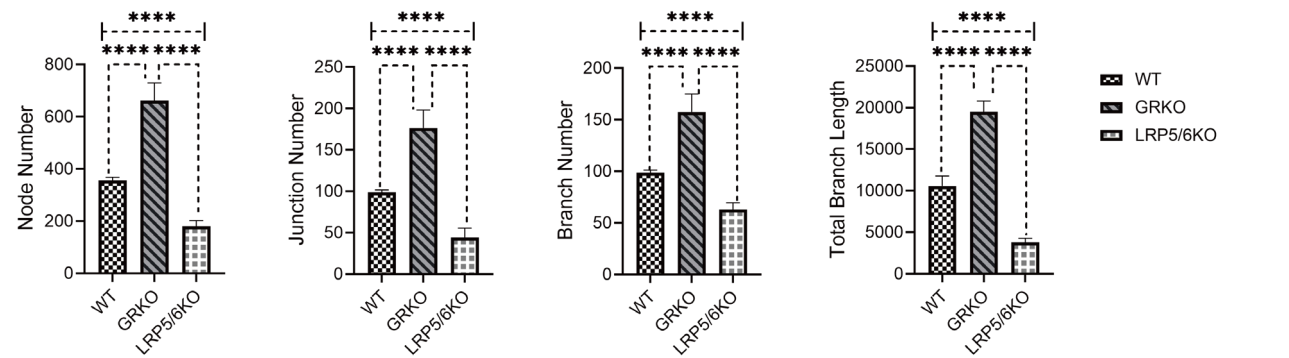

Supplement: Supplementary file 2 — Supplementary material 2 (PDF 801 kb) [file 10456_2021_9773_MOESM2_ESM.pdf]

A

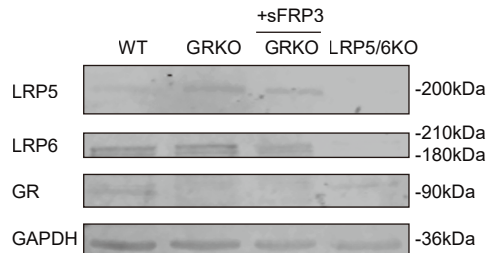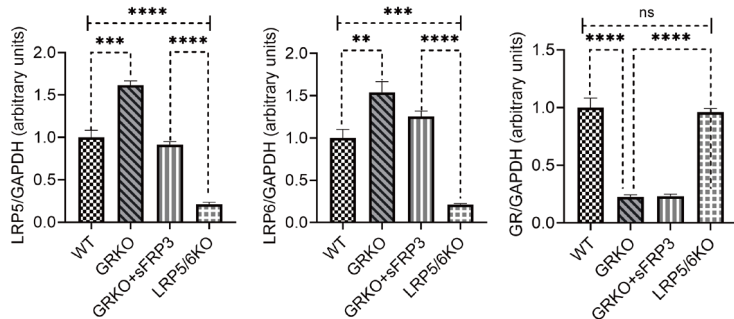

B

## Cell Viability Assay

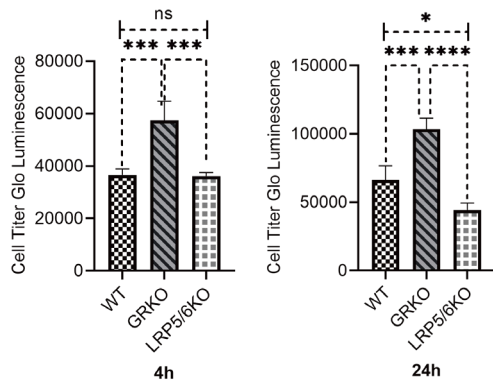

C

## Cell Proliferation Assay

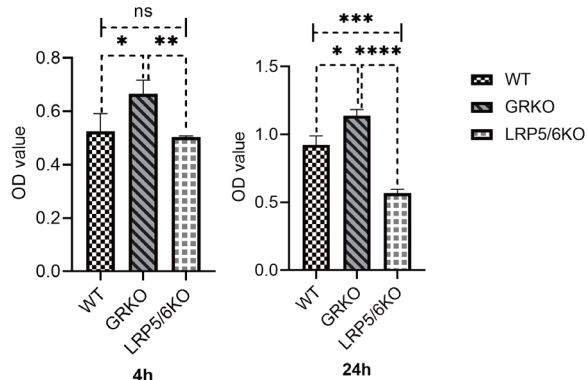

Supplement: Supplementary file 3 — Supplementary material 3 (PDF 462 kb) [file 10456_2021_9773_MOESM3_ESM.pdf]

A

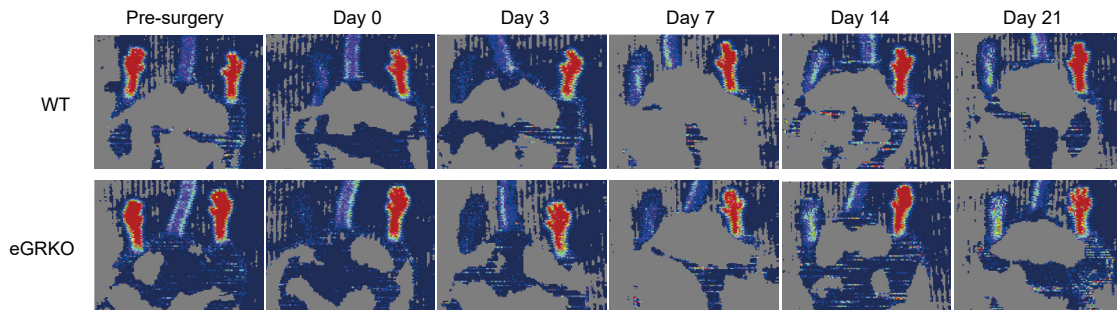

B

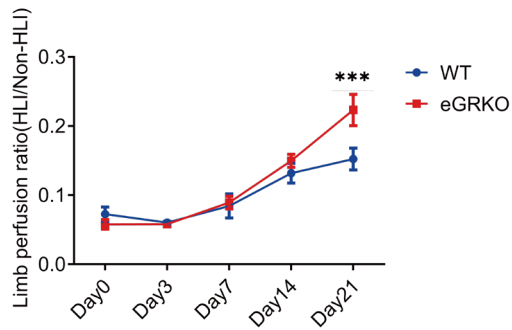

C

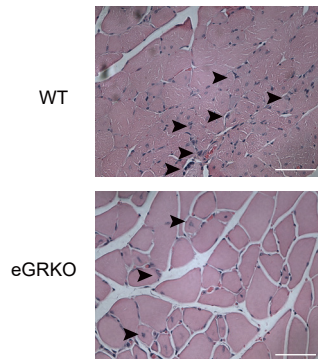

Supplement: Supplementary file 4 — Supplementary material 4 (PDF 641 kb) [file 10456_2021_9773_MOESM4_ESM.pdf]

**A**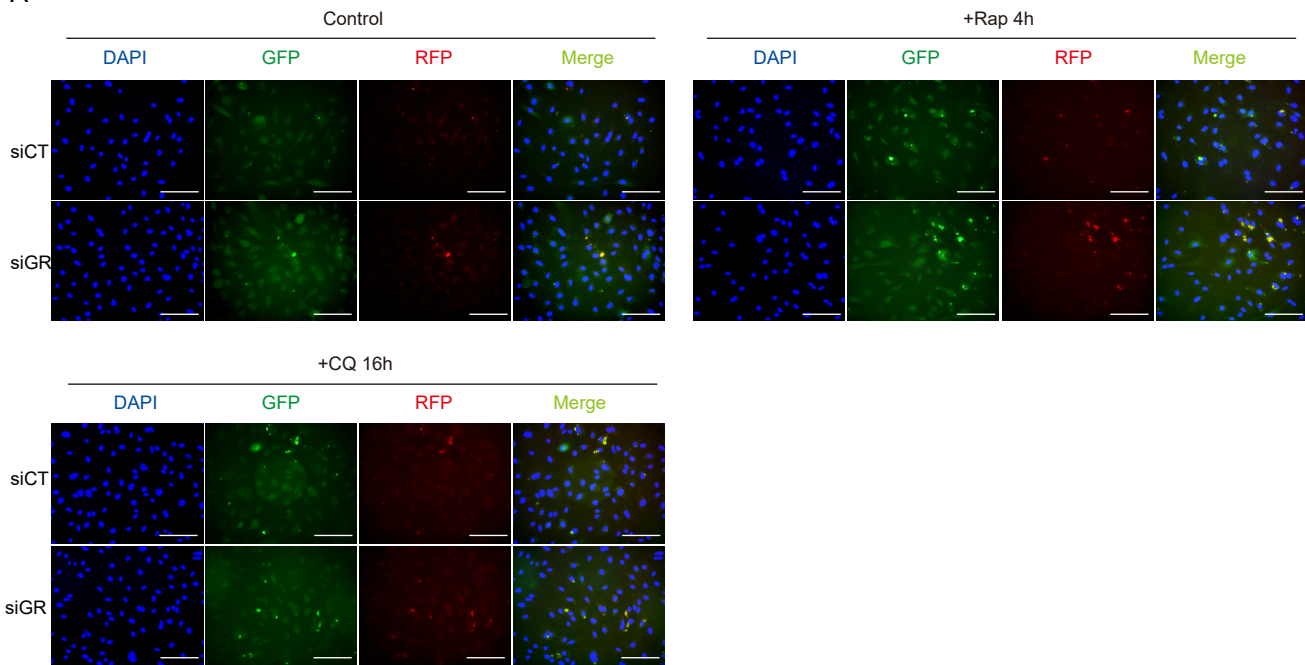**B**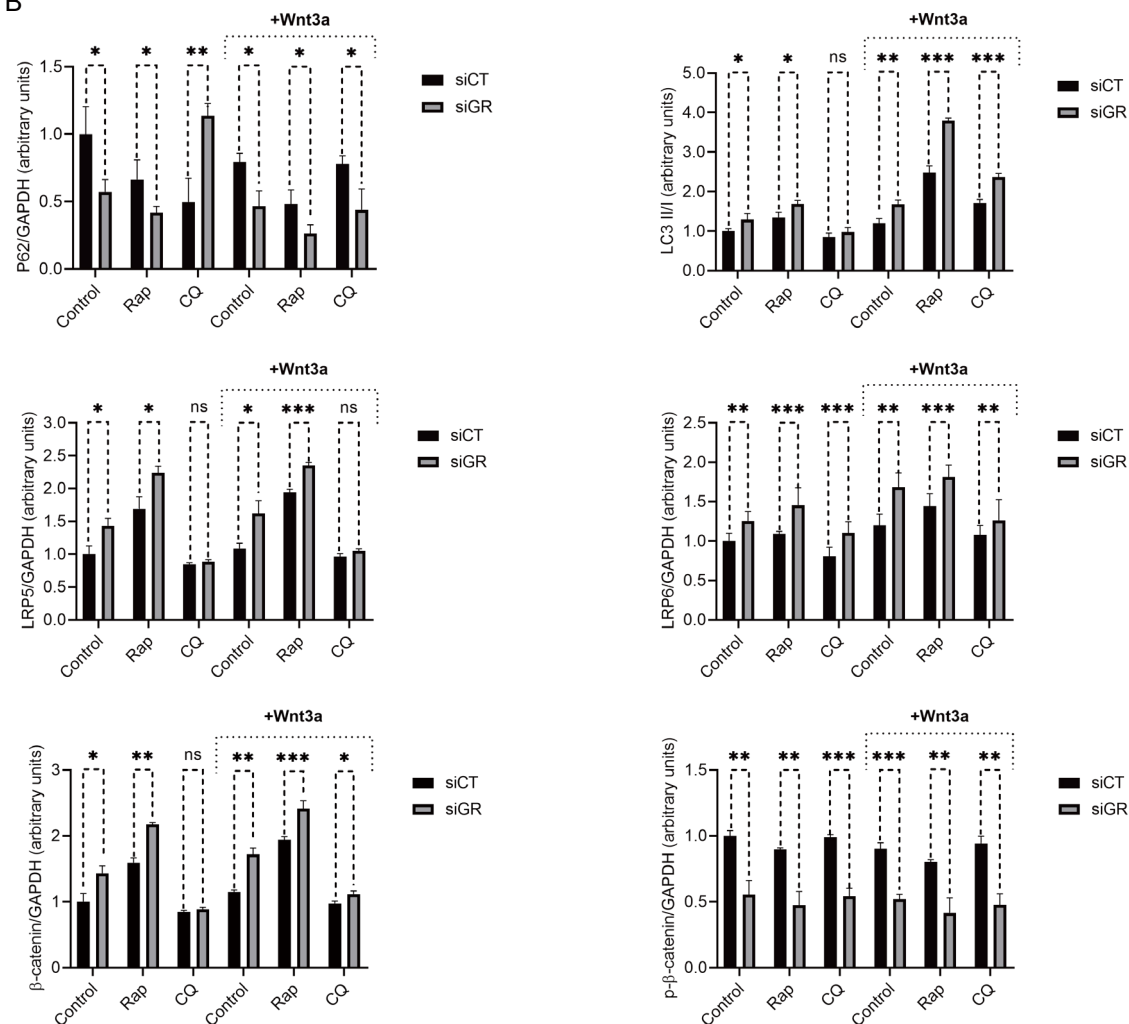

Supplement: Supplementary file 5 — Supplementary material 5 (PDF 836 kb) [file 10456_2021_9773_MOESM5_ESM.pdf]
