# Supplementary material for: Loss of endothelial glucocorticoid receptor promotes angiogenesis via upregulation of Wnt/β-catenin pathway
Source: Angiogenesis. 2021 Mar 2;24(3):631–45. doi: 10.1007/s10456-021-09773-x (PMC8292305; doi:10.1007/s10456-021-09773-x)
Supplement: Supplementary file 6 — Supplementary material 6 (DOCX 16 kb) [file 10456_2021_9773_MOESM6_ESM.docx]

**Supplementary Fig. 1 Loss of GR induces accumulation of β-catenin in the nucleus.**

**(a)** Representative images of MLECs stained with antibodies to β-catenin and GR (scale bar, 50 µm). **(b)** Quantitative results indicate loss of GR induces the accumulation of β-catenin in the nucleus (t-test) (n=5/group). siCT: MLECs transfected with control siRNA; GRsi: MLECs transfected with control siRNA.

**Supplementary Fig. 2 Tube formation assay in MAOECs from different genotypes.**

**(a)** Representative tube formation images and tube maps generated by the software Image J-Angiogenesis Analyzer from WT MAOECs or GRKO MAOECs under different conditions. **(b)** Quantification of images in A. In MAOECs, GRKO cells had more nodes, junctions and branches than WT cells (t-test). Administration of Wnt3a and sFRP3 regulated tube formation in opposite manners (One-way ANOVA) (n=5/group). **P<0.05, ***P<0.001, ****P<0.0001*. **(c)** Representative tube formation images and tube maps generated by the software Image J-Angiogenesis Analyzer from WT MAOECs, GRKO MAOECs or LRP5/6KO MAOECs. **(d)** eLRP5/6KO MAOECs had fewer nodes, junctions and branches in vitro compared to cells from both WT or eGR KO conditions (t-test) (n=5/group, *****P<0.0001*). WT: primary MAOECs from WT mice; GRKO: primary MAOECs from eGRKO mice; LRP5/6KO: primary MAOECs from eLRP5/6KO mice.

**Supplementary Fig. 3 Loss of endothelial LRP5/6 suppresses cell viability and proliferation in vitro.**

**(a)** Representative Western blot and corresponding densitometry of primary MAOECs isolated from eGRKO mice, eLRP5/6KO mice and their WT littermates. **(b, c)** Loss of GR promoted cell viability and proliferation at either 4h or 24h in MAOECs, while cell viability and proliferation was suppressed in eLRP5/6KO MAOECs (t-test, n=3/group, **P<0.05, **P<0.01, ***P<0.001, ****P<0.0001, ns not significant*). WT: primary MAOECs from WT mice; GRKO: primary MAOECs from eGRKO mice; LRP5/6KO: primary MAOECs from eLRP5/6KO mice.

**Supplementary Fig. 4** **Angiogenesis is enhanced in endothelial GR deficient mice** **after hind limb ischemia.**

**(a)** Representative Laser Doppler images and **(b)** corresponding quantification of limb perfusion ratio (HLI/Non-HLI) show that from the 7^th^ day after surgery, the blood flow in eGRKO mice recovered better than that of WT littermates *(n=3/group, ***P<0.001).* **(c)** Representative hematoxylin and eosin (H&E)-stained sections of ischemic muscles from WT and eGRKO mice at days 21 post surgery. Scale bar: 100 μm. Black arrowheads indicate the necrotic myocytes.

**Supplementary Fig. 5 Loss of GR up-regulates Wnt/β-catenin pathway by enhancing autophagy flux.**

**(a)** Representative images of autophagosomes (yellow) and autophagolysosomes (red) in MLECs transfected with RFP-GFP-LC3 for 16 h, treated with rapamycin (4h) or chloroquine (16h) (scale bar, 100 µm). **(b)** Western blot densitometry of Rap/CQ treatment and Wnt3a/Rap/CQ treatment conditions (t-test) (n=3/group). After additional treatment with Wnt3a, the expression of P62 decreased and the LC3 II/I conversion was enhanced. Rap: rapamycin; CQ: chloroquine. **P<0.05, **P<0.01, ***P<0.001, ****P<0.0001, ns not significant*.
